# Evaluation of a multiview architecture for automatic vertebral labeling of palliative radiotherapy simulation CT images

**DOI:** 10.1002/mp.14415

**Published:** 2020-09-15

**Authors:** Tucker J. Netherton, Dong Joo Rhee, Carlos E. Cardenas, Caroline Chung, Ann H. Klopp, Christine B. Peterson, Rebecca M. Howell, Peter A. Balter, Laurence E. Court

**Affiliations:** ^1^ Department of Radiation Physics The University of Texas MD Anderson Cancer Center Houston TX 77030 USA; ^2^ The University of Texas MD Anderson Graduate School of Biomedical Science Houston TX 77030 USA; ^3^ Department of Radiation Oncology The University of Texas MD Anderson Cancer Center Houston TX 77030 USA; ^4^ Department of Biostatistics The University of Texas MD Anderson Cancer Center Houston TX 77030 USA

**Keywords:** automatic vertebral labeling, deep learning

## Abstract

**Purpose:**

The purpose of this work was to evaluate the performance of X‐Net, a multiview deep learning architecture, to automatically label vertebral levels (S2‐C1) in palliative radiotherapy simulation CT scans.

**Methods:**

For each patient CT scan, our automated approach 1) segmented spinal canal using a convolutional‐neural network (CNN), 2) formed sagittal and coronal intensity projection pairs, 3) labeled vertebral levels with X‐Net, and 4) detected irregular intervertebral spacing using an analytic methodology. The spinal canal CNN was trained via fivefold cross validation using 1,966 simulation CT scans and evaluated on 330 CT scans. After labeling vertebral levels (S2‐C1) in 897 palliative radiotherapy simulation CT scans, a volume of interest surrounding the spinal canal in each patient's CT scan was converted into sagittal and coronal intensity projection image pairs. Then, intensity projection image pairs were augmented and used to train X‐Net to automatically label vertebral levels using fivefold cross validation (*n* = 803). Prior to testing upon the final test set (*n* = 94), CT scans of patients with anatomical abnormalities, surgical implants, or other atypical features from the final test set were placed in an outlier group (*n* = 20), whereas those without these features were placed in a normative group (*n* = 74). The performance of X‐Net, X‐Net Ensemble, and another leading vertebral labeling architecture (Btrfly Net) was evaluated on both groups using identification rate, localization error, and other metrics. The performance of our approach was also evaluated on the MICCAI 2014 test dataset (*n* = 60). Finally, a method to detect irregular intervertebral spacing was created based on the rate of change in spacing between predicted vertebral body locations and was also evaluated using the final test set. Receiver operating characteristic analysis was used to investigate the performance of the method to detect irregular intervertebral spacing.

**Results:**

The spinal canal architecture yielded centroid coordinates spanning S2‐C1 with submillimeter accuracy (mean ± standard deviation, 0.399 ± 0.299 mm; *n* = 330 patients) and was robust in the localization of spinal canal centroid to surgical implants and widespread metastases. Cross‐validation testing of X‐Net for vertebral labeling revealed that the deep learning model performance (F_1_ score, precision, and sensitivity) improved with CT scan length. The X‐Net, X‐Net Ensemble, and Btrfly Net mean identification rates and localization errors were 92.4% and 2.3 mm, 94.2% and 2.2 mm, and 90.5% and 3.4 mm, respectively, in the final test set and 96.7% and 2.2 mm, 96.9% and 2.0 mm, and 94.8% and 3.3 mm, respectively, within the normative group of the final test set. The X‐Net Ensemble yielded the highest percentage of patients (94%) having all vertebral bodies identified correctly in the final test set when the three most inferior and superior vertebral bodies were excluded from the CT scan. The method used to detect labeling failures had 67% sensitivity and 95% specificity when combined with the X‐Net Ensemble and flagged five of six patients with atypical vertebral counts (additional thoracic (T13), additional lumbar (L6) or only four lumbar vertebrae). Mean identification rate on the MICCAI 2014 dataset using an X‐Net Ensemble was increased from 86.8% to 91.3% through the use of transfer learning and obtained state‐of‐the‐art results for various regions of the spine.

**Conclusions:**

We trained X‐Net, our unique convolutional neural network, to automatically label vertebral levels from S2 to C1 on palliative radiotherapy CT images and found that an ensemble of X‐Net models had high vertebral body identification rate (94.2%) and small localization errors (2.2 ± 1.8 mm). In addition, our transfer learning approach achieved state‐of‐the‐art results on a well‐known benchmark dataset with high identification rate (91.3%) and low localization error (3.3 mm ± 2.7 mm). When we pre‐screened radiotherapy CT images for the presence of hardware, surgical implants, or other anatomic abnormalities prior to the use of X‐Net, it labeled the spine correctly in more than 97% of patients and 94% of patients when scans were not prescreened. Automatically generated labels are robust to widespread vertebral metastases and surgical implants and our method to detect labeling failures based on neighborhood intervertebral spacing can reliably identify patients with an additional lumbar or thoracic vertebral body.

## 1 INTRODUCTION

When cancer metastasizes to the spine, it can cause debilitating symptoms, such as severe bone pain, pathologic fracture, and spinal cord compression.^1^ Such symptoms can be effectively alleviated or controlled with palliative radiotherapy.^2^, ^3^, ^4^ Prior to radiotherapy, it is necessary to identify the involved vertebral levels. To properly label vertebral levels, anatomical landmarks such as the clavicle, xiphoid process, and sternal angle provide information regarding the identity of each unique rib and vertebra and can be visualized using CT.^5^ Vertebral levels are verified on radiotherapy CT scans by a radiation oncologist during treatment planning as well as by a therapist during treatment via imaging guidance. In any of these instances, labeling vertebral levels can be challenging when variations in anatomy, surgical implants, or structural degradations due to metastatic disease are present.

Labeling vertebral levels in CT scans affect both treatment planning and the treatment itself. Accuracy and time efficiency of vertebral labeling are essential to ensuring that the correct target is treated and that the patient care path is expedited, as delays in the patient care path can lead to delays in symptom relief.^6^, ^7^ This task and many others are performed during treatment planning and can be time‐consuming, as input from different clinical experts is required at multiple stages.^8^ Because recent advances in deep learning have resulted in time reductions and expert‐level performance when applied to medical image‐based tasks^8^, ^9^, ^10^, ^11^, we applied convolutional neural networks (CNNs) to the CT‐based task of vertebral labeling. Our purpose was to design an approach to automatically label vertebral levels in radiotherapy simulation CT scans with high labeling accuracy and small localization errors. This will help us achieve our long‐term goals of automating radiotherapy treatment planning, reducing treatment planning time, decreasing the likelihood of vertebral level labeling errors, and increasing the efficiency of the patient care path in palliative radiotherapy.

Thus far, no reported studies have used deep learning to label vertebral levels for the purposes of automatic radiotherapy treatment planning. Our approach uses X‐Net, inspired by a combination of the Btrfly Net^12^ and VNet^13^ architectures, and is a multiview architecture for automatic vertebral labeling that is end‐end trainable. Furthermore, our data are curated specifically for palliative radiotherapy treatment planning and include simulation CT scans with large fields of view (containing the entire patient body), surgical implants, variable scan lengths, and numerous spinal metastases. In the present study, we investigated the performance of our vertebral labeling architecture upon palliative radiotherapy simulation CT scans as well as a well‐known diagnostic CT benchmark dataset from the MICCAI 2014 CSI workshop.^14^ Our approach (a) applied a CNN to segment spinal canal as an essential preprocessing step, (b) formed sagittal and coronal intensity projection image pairs from a volume of interest surrounding the spinal canal, (c) used X‐Net, an X‐Net Ensemble, and Btrfly Net (for comparison) to label vertebral levels, and (d) implemented a methodology to detect irregular intervertebral spacing.

## 2 MATERIALS AND METHODS

### 2.A X‐Net Architecture

**Fig. 1:**
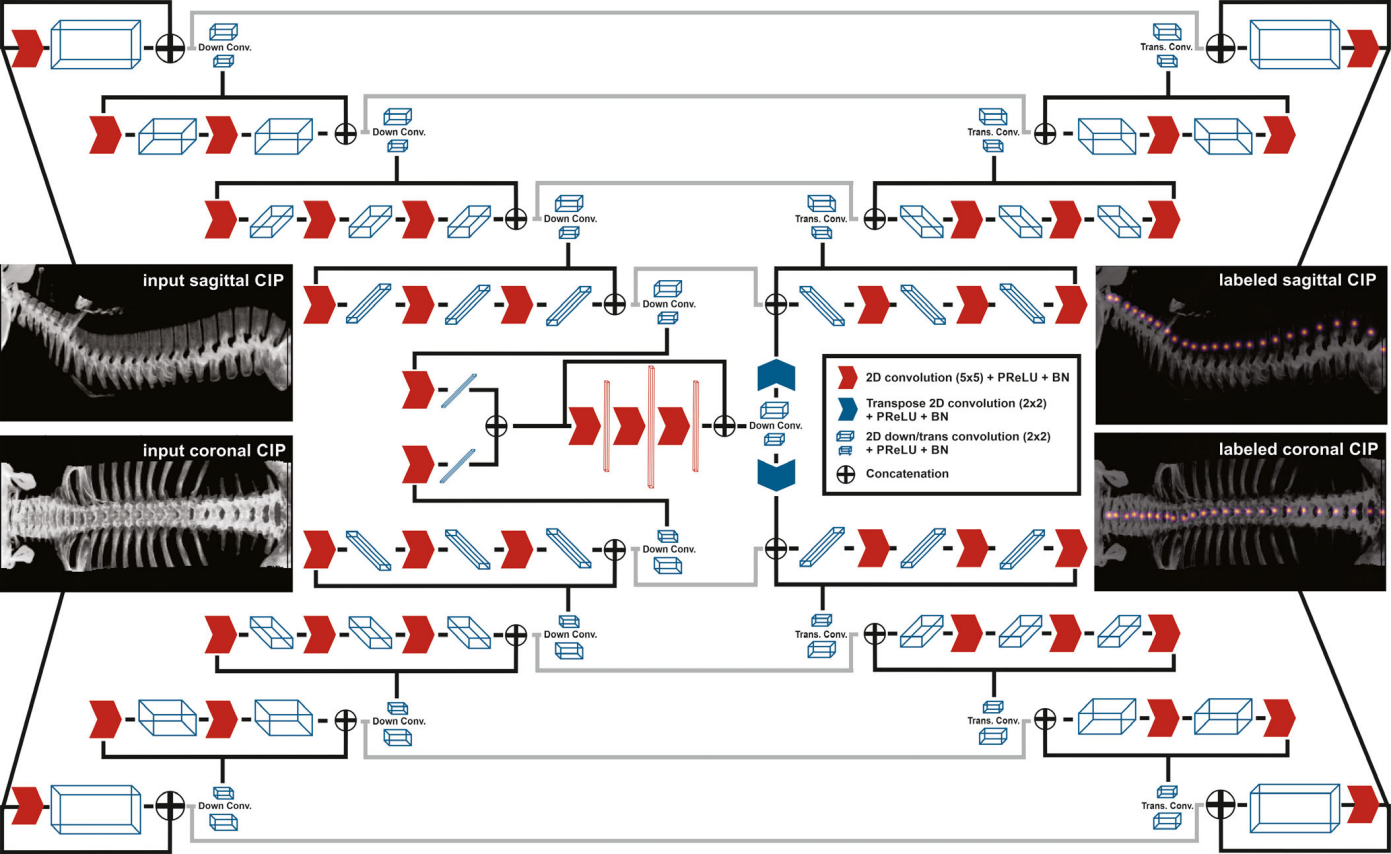
The X‐Net architecture. Input arms receive a sagittal and coronal intensity projection image pair and output sagittal and coronal arrays containing a unique channel for each vertebral label. Red arrows indicate 2D convolution**s**, PReLU activation, and batch norm (BN) layers. Blue arrows indicate transpose operations. White and blue rectangular volumes depict the general shape of the feature space after each operation. CIP, custom intensity projection. [Color figure can be viewed at wileyonlinelibrary.com]

The architecture of X‐Net is shown in Fig. 1. X‐Net is “X‐shaped” and uses fewer computational resources than other 3D approaches since two planar projection images are input to the network instead of a 3D volume per patient scan. Inspired by a study performed by Sekuboyina et al.,^12^ input arms intake sagittal and coronal intensity projection images constructed from the patient CT scan. X‐Net is a fully convolutional network (FCN) with four encoding stages and four decoding stages before layers from both arms are concatenated together. In addition, our architecture uses PreLU activations and excludes the use of max‐pooling layers, up‐sampling layers, and dropout. Residual blocks are incorporated at each stage. All kernels are 5 × 5 except for down and transpose convolutions, which are 2 × 2. Output filters are held constant in the first four stages (32, 64, 128, 256) except for the dense feature space shared by both arms of the architecture which has filters of 512,1024, and 512 (depicted by vertical red rectangular prisms in Fig. 1). In the last convolutional stage before decoding, X‐Net feature maps are concatenated and shared by the sagittal and coronal arms of the network. X‐Net outputs a 608 × 192 × 27 array per arm and predicts a 2D Gaussian label for each channel. Twenty‐seven output channels in the final convolutional layers provide a latent space for 24 vertebral bodies, 2 sacral bones, and 1 null channel for background before final activation by a sigmoid function.

### 2.B Datasets

All CT scans used in Table I are radiotherapy simulation CT scans have field‐of‐views which encompass the entire patient. Spinal canal segmentation cases include both CT scans and clinical spinal canal/cord contours. Vertebral body labeling cases include both CT scans and vertebral level contours (C1‐S2). Testing data from the vertebral body labeling task do not overlap with the training data from the canal segmentation cohort.

**Table I:** Imaging cohorts used in the training and testing of canal segmentation, labeling, and failure detection tasks.

	Canal Segmentation	Vertebral Body Labeling	Irregular Intervertebral Spacing Detection
Cross validation	1,966	803	162
Final Testing	330	94	94
Total	2,296	897	n/a

#### 2.B.1 Simulation CTs: spinal canal segmentation

Patient data were retrospectively acquired from a database containing radiotherapy simulation CT scans and used for training and testing our spinal canal segmentation tool. Specifically, 2,296 CT scans (Table I) from our institution were from patients who received radiotherapy to various cancers in the body (esophagus, spine, and head and neck). The CT scans were a combination of contrast and noncontrast scans as well as those with and without surgical implants in the spine. Median pixel size was 1.170 (minimum = 0.754, maximum = 1.37) and median slice thickness was 2.5 mm (minimum = 0.4 mm, maximum = 5.0 mm). Clinical contours of spinal cord and spinal canal from CT scans of patients who were retrospectively treated with radiation therapy were used for the training and testing of the spinal canal segmentation model.

#### 2.B.2 Simulation CTs: vertebral body labeling

Radiotherapy simulation CT scans of variable scan length were also used to train and test our vertebral level labeling solution. For this purpose, 897 simulation CT scans of patients who received radiotherapy to the spine were obtained. These CT scans spanned three CT vendors: Phillips, Siemens, and GE. Median pixel size was 1.170 (minimum = 0.683, maximum = 1.365) and median slice thickness was 2.5 mm (minimum = 1.0 mm, maximum = 5.0 mm). Median field‐of‐view was 600 mm (minimum = 350 mm, maximum = 699 mm). The images contained a combination of contrast and noncontrast scans.

For final testing of vertebral body labeling (*n = *94), patient CT scans (Table I) were placed in two groups (normative group and outliers). These two groups (normative, *n = *74; outlier, *n* = 20) were formed based on our hypothesis that a normative patient group would yield the best performance based on the fact that outlier features can occlude valuable anatomic features used to identify vertebral level.

The outliers (*n* = 20) consisted of those with surgical implants (*n* = 6), abnormal vertebral anatomy (*n* = 6), a prone setup (*n* = 1), numerous surgical clips (*n* = 1), pacemakers (*n* = 2), an inclined setup (*n* = 1), a vertebroplasty (*n* = 1), myelogram (*n* = 1), and a pediatric patient (*n* = 1). Patients having titanium surgical implants and/or vertebroplasties in these datasets ranged the length of the spine. Intravenous contrast within the aorta was often visible anterior to the lumbar spine in these datasets. Patients in the normative group (*n* = 74) were randomly selected from a large cohort of patients (*n* = 803) who did not have the outlier features listed above.

#### 2.B.3 Diagnostic CTs: vertebral body labeling

The MICCAI 2014^15^ dataset is a publicly available dataset composed of tightly cropped (spine‐focused) diagnostic CT scans of 125 patients with various pathologies. In total are 302 scans (training = 242, testing = 60) complete with spinal centroid annotations. Featured in Table II is the testing portion of this cohort. Median pixel size was 0.332 (minimum = 0.227, maximum = 0.508) and median slice thickness was 2.5 mm (minimum = 0.625 mm, maximum = 2.5 mm). Median field‐of‐view was 170 mm (minimum = 116 mm, maximum = 260 mm).

**Table II:** Number of cases and vertebral counts used for the cross validation and testing of vertebral labeling.

	Cross Validation Data	Final Testing	MICCAI 2014*
Cases	803	94	60
Cervical	13,736	264	191
Thoracic	39,936	746	321
Lumbar	17,253	285	113
Sacral	4,990	71	32

* The MICCAI 2014^1^ testing data from the CSI workshop.

### 2.C Preprocessing

#### 2.C.1 Intensity projection formation

**Fig. 2:**
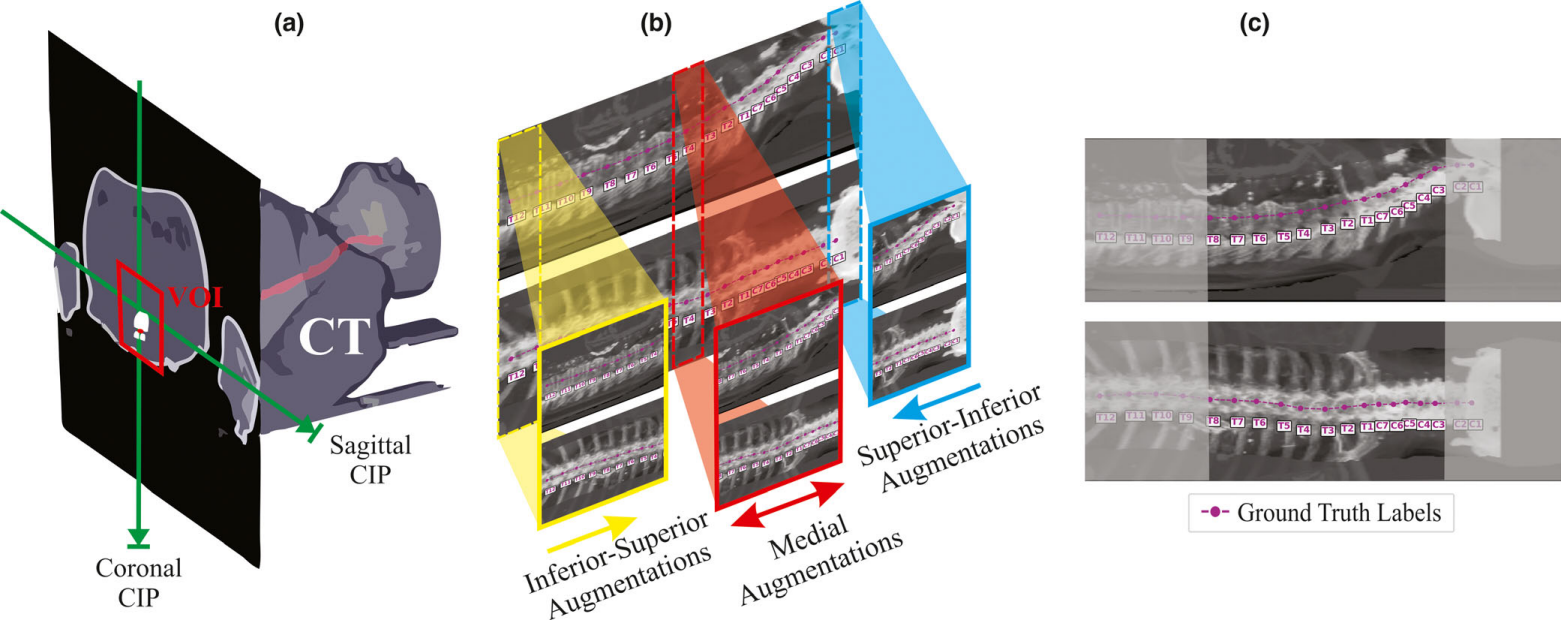
Visual depiction of IP image pair formation. (a) A 3D diagram depicting the formation of the coronal and sagittal intensity projection images across the VOI (red rectangle shows cross section of VOI) centered about the spinal canal (red). Maximum pixel values or mean pixel values are projected across orthogonal directions to obtain the MIP and AIP. (b) A depiction of how intensity projection pairs are augmented for training and for ablative testing. (c). An example of a central medial augmentation taken from the central region of an intensity projection pair (grey regions are removed) with ground truth annotations in magenta. [Color figure can be viewed at wileyonlinelibrary.com]

When sagittal and coronal intensity projection images are created from CT scans, ribs and other high‐intensity pixel values (from surgical implants or intravenous contrast) can occlude bony anatomy and important anatomic information. Since the removal of the ribs has been demonstrated to improve localization performance, and also since different intensity projection images (average intensity projection (AIP) vs maximum intensity projection (MIP)) yield different results when used in vertebral body localization,^16^ we created custom intensity projection (CIP) images, denoted as **x** equation below. Thus,

$$x_{\mathrm{view}}=wAIP_{\mathrm{VOI}}+MIP_{\mathrm{VOI}}+w^{‐2}MIP_{\tilde{\mathrm{VOI}}},$$

where **x** (sagittal or coronal CIP) is formed from the AIP and MIP images. Also, of note, is that the volume of interest (VOI) denotation [Fig. 2(a)] indicates that CIPs were formed within a cropped VOI ( 55 pixels x 55 pixels x spinal canal length) from the patient CT. This VOI is made using the spinal canal segmentation and is featured in [Fig. 2(a)]. Regions *outside* the VOI are denoted $\tilde{\mathrm{VOI}}$. The scalar factor *w* was set to *w = *2 to best visualize intervertebral spaces; its value was based on a visual inspection of CT scans of various patients with surgical implants and intravenous contrast. Before training, 48 CIP augmentations were generated by applying the formula above to each CT scan by varying upper (800–1600) and lower (−1000 to −200) CT number normalization windows by an increment of 200. An additional 48 CIP augmentations with these same intensity normalizations were also created to have constrained VOIs to contain only the vertebral bodies (i.e., cut to contain regions anterior to the centroid of the spinal canal). This removed posterior vertebral processes and allowed for improved visualization of intervertebral spaces on coronal projection images. Thus, per patient, 96 intensity augmentations were formed. The optimal combination of intensity augmentations was determined by combining 1, 2, 3, 4, 7, 16, and 96 CIP image pairs and examining X‐Net performance (e.g., identification rate, localization error) upon the cross‐validation dataset. All data were augmented by cropping every IP image pair in 25‐mm increments from the superior to inferior direction of the CT scan, inferior to superior direction of the CT scan, and from the middle to distal ends of the CT scan [Figs. 2(b)‐2(c)]. This cropping was performed only if a vertebral body was present within the portion of the CT scan that was cropped. MIP images were also created and augmented in the same fashion in order to evaluate X‐Net performance for MIP‐ vs CIP‐based training and testing.

#### 2.C.2 Ground truth vertebral label construction

All vertebral level centroids were hand‐labeled by a team of three research assistants trained by a qualified medical physicist using treatment planning software. All vertebral levels were checked once by a separate research assistant and approved by the medical physicist. All vertebrae from 897 simulation CT scans were labeled by placing separate spherical contours at the centroid of each vertebral body per vertebral level (L5‐C1). In addition, spherical contours were placed at S1 midline between the sacral promontory and sacral canal and also at S2 at the intersection of the left‐right sacroiliac joint line with the line bisecting the sacral foramina.

All CT images were interpolated (spline, order = 1) to the median slice thickness (2.5 mm) before IP image formation. All data from the MICCAI 2014 cohort were resized and padded to have pixel sizes and fields‐of‐view within range (e.g., 1.17 mm × 1.17 mm, 512 × 512 pixels) of the radiotherapy simulation CT data. Vertebral body centroid coordinates, ξ, were obtained by projecting the coordinates of each spherical contour centroid into the plane of the IP image. IP images were cropped around the VOI from the spinal canal segmentation and padded to *l = 608* and *w = 192*. This padding approach allows for scans of any length up to 60.3 cm in length (which is larger than any CT scan in our dataset). For the *i^th^* channel in each IP image label, y_i_, a 2D Gaussian distribution denoted the ground truth label as.

$$y_{i}=\frac{1}{\sigma \sqrt{2\pi }}e^{‐{\left|\left|\xi ‐\xi_{i}\right|\right|}^{2}/\left(2\sigma^{2}\right)},$$

where standard deviation (here *σ = 2*) controls the extent of the Gaussian spread. The dimensions of ***x*** *_view_* and ***y*** *_view_* label arrays were $x_{\mathrm{view}}\in R^{608x192}$ and $y_{\mathrm{view}}\in R^{608x192x27}$, respectively, with the 27th channel constituting a null channel for background. After CT number normalization, sagittal and coronal label arrays were normalized to 225 intensity levels, cropped at a pixel value of 75, and converted to Boolean arrays. The threshold of 75 was chosen so that the Boolean labels did not overlap adjacent vertebral bodies.

### 2.D Metrics for vertebral level labeling

To assess vertebral labeling performance, class‐ and patient‐specific metrics were examined. Here, each k^th^ class referred to 1 of 26 channels used to distinguish each vertebral body. Two important metrics for vertebral labeling are labeling accuracy and localization error. The coordinates of the ground truth labels are represented as $\xi_{i}\in R^{3}\left(i\in \left\{1,2,\ldots ,26\right\}\right)$, with *i* denoting the *i^th^* vertebral or sacral body. A vertebral body was considered to be correctly identified if the following criteria were satisfied (same approach as used in^14^, ^15^, ^16^, ^17^, ^18^, ^19^, ^20^, ^21^, ^22^):

1. The 3D Euclidean distance from the ground truth to the predicted vertebral body location (localization error) is less than 20 mm;
2. The predicted vertebral body is the closest vertebral body to the ground truth.

Coordinates from sagittal and coronal predictions of vertebral label were combined to yield their 3D label coordinates. If all criteria were satisfied, the vertebral body was scored as a true positive (TP). If the model predicted the presence of a vertebral body when there should not have been one *or* the vertebral body failed to meet any of the criteria, the vertebral body was scored as a false positive (FP). True negatives (TNs) were defined as correctly identified null predictions (correct absence of vertebral bodies), and false negatives (FNs) were defined as null predictions when corresponding vertebral bodies were actually present.

Patient‐specific metrics calculated for every *j* ^th^ patient across *i^th^* $\in \left(1\ldots n_{j}\right)$ vertebrae, where *n_j_* is the total number of vertebrae present in every *j_th_* patient, were:

1. Localization error:$R_{j}=\sqrt{{\left(\xi_{GT,j}‐\xi_{P,j}\right)}^{2}}$, where ξ represents a vector of 3D location
2. Rate of change of spacing: $\delta_{j}=\left|S_{i+1}‐S_{i}\right|$, where S_j_ is a given vertebral space $\xi_{j,i+1}‐\xi_{j,i}$
3. Patient accuracy: $A_{j}=\frac{1}{n_{j}}\sum_{i=1}^{n_{j}}TP_{i,j}$
4. Patient localization error: $E_{j}=\frac{1}{n_{j}}\sum_{i=1}^{n_{j}}R_{i,j}$
5. Patient precision; $P_{j}=\frac{1}{n_{j}}\sum_{i=1}^{n_{j}}TP_{i,j}/\left(TP_{i,j}+FP_{i,j}\right)$
6. Patient sensitivity: $S_{j}=\frac{1}{n_{j}}\sum_{i=1}^{n_{j}}TP_{i,j}/\left(TP_{i,j}+FN_{i,j}\right)$
7. F_1_‐score: $F1_{j}=2\left(S_{i,j}\times P_{i,j}\right)/\left(S_{i,j}+P_{i,j}\right)$

Class‐specific metrics, calculated for every k^th^ vertebral body, were:

1. Identification rate: $IR_{k}=\frac{1}{m_{k}}\sum_{k=l}^{n_{k}}TP_{k}$, where *m* is the total number of *k_th_* ground truth vertebrae in *all* patient CT images
2. Class localization error: $E_{k}=\frac{1}{m_{k}}\sum_{k=l}^{n_{k}}\sqrt{{\left(\xi_{GT,k}‐\xi_{P,k}\right)}^{2}}$

By selecting *k* ∈ (1…7), (8…19), (20…24), (24…26), and (1…26), class‐specific metrics were reported for the cervical, thoracic, lumbar, sacral, and all spinal regions, respectively.

### 2.E Experiments

For all training and inference tasks, tensorflow^23^ and a DGX workstation with a 16‐GB NVIDIA V100 graphics processing unit was used.

#### 2.E.1 Training a CNN for spinal canal segmentation (preprocessing)

Due to the curvature of the rib cage, vertebral bodies in 2D MIP images can be shadowed by ribs and decrease vertebral labeling accuracy.^23^ One method to crop the ribs from the CT scan volume is to crop around a fixed distance from the spinal canal, creating a volume of interest for subsequent experiments described in this work. Thus, the FCN‐8s^24^ with batch‐normalization layers added (identical to that described by Rhee et al.^25^) was trained to segment spinal canal. Dice loss and Adam optimizer were used during training. Our training strategy used fivefold cross‐validation on data in Table I. For each image slice, the 2D distance between the predicted spinal canal centroid and the ground truth spinal canal centroid was used to evaluate canal segmentation performance.

#### 2.E.2 Training and cross validation for vertebral level labeling using simulation CT images

For this experiment, 803 patient simulation CT images with labeled vertebral bodies were used to train X‐Net using fivefold cross‐validation. The top performing data split was also trained using Btrfly Net; loss functions, and choice of optimizer were the same for Btrfly Net. The process for CIP and MIP image generation was also the same for Btrfly Net. The total loss function was computed by taking the sum of losses from both arms of X‐Net (or Btrfly Net) as follows:

$$Loss_{\mathrm{total}}=Loss_{S}+Loss_{C};$$

$$Loss_{\mathrm{view}}=Dice\left(Y,\overset{ˇ}{Y}\right)+CE\left(Y,\overset{ˇ}{Y}\right);$$

where view∈ {sagittal, coronal} represents planar intensity projection images, *Dice* ^26^ is the Dice similarity coefficient that influences degree of overlap of predicted and ground truth labels, *CE* is the cross‐entropy function that provides additional global regularization, and *Y* and $\overset{ˇ}{Y}$ are the ground truth labels and predicted labels, respectively. A batch size of eight CIP image pairs and learning rate 2 x 10^‐4^ was used during training.

Using the metrics from section 2.D., the vertebral labeling model with the best performance from fivefold cross‐validation was selected for subsequent testing using the final test set of patients (*n* = 94). In addition, metrics for MIP‐ vs CIP‐based training and testing were compared to evaluate whether choice of IP influences model performance.

#### 2.E.3 Study of model performance vs CT scan length

To determine how model performance changes as a function of CT scan length, an ablative study was performed using the testing data from the highest performing cross validation split. Using the image augmentation strategy described in section 2.C.1, IP image pairs were cropped and fed into the top performing model. F_1_‐score, specificity, sensitivity, and were recorded for each cropping and reported as a function of the number of vertebral bodies within each cropping.

#### 2.E.4 Optimizing the detection of irregularly spaced vertebral bodies

To detect irregularly spaced vertebral bodies, a post processing technique examining vertebral spacing was developed using the best performing model from the cross‐validation dataset. This technique is important, as its goal was to flag incorrect vertebral labeling results from the vertebral labeling model and serve as a means automatic quality assurance. For each patient, the distance between each adjacent predicted vertebral body center was recorded. Then, the rate of change in spacing (δ) was recorded for each patient. This δ was recorded for each patient CT scan using the predicted 3D coordinates of vertebral levels as well as the sagittal CIP image coordinates. A vertebral level prediction on any given patient was considered to have irregular intervertebral spacing if the 3D, sagittal, or coronal δ exceeded a scalar threshold. The ability of this technique to flag patients with suspicious vertebral spacing after the prediction of vertebral levels was optimized using receiver operating characteristics (ROC) analysis.

#### 2.E.5 Final testing of vertebral level labeling and irregular spacing detection on radiotherapy simulation CT scans

The best performing model using X‐Net and an X‐Net Ensemble (of all five cross‐validation CIP or MIP models) were tested by predicting vertebral levels on 94 patients in the final test set. Metrics from section 2.D. were used to evaluate model performance. Vertebral labeling results from Btrfly Net were also provided for reference using the same testing data. The same patient split and CIP images were used to train Btrfly Net as was X‐Net. Any δ's exceeding a threshold were flagged for review to catch labeling failures due to irregularly spaced vertebral centroid predictions. Sensitivity and specificity of this detection method were recorded and individual failure cases were examined.

#### 2.E.6 Comparison to previous work

The performance of X‐Net on a separate imaging cohort, described in section 2.B.3, was evaluated through two approaches. The first approach trained multiple X‐Net models using the training cohort (*n* = 242) and evaluated the models on the testing cohort (*n* = 60) using an ensemble of three X‐Net models (all trained separately from scratch). The second approach applies transfer learning using the five models from cross‐validation in section 2.E.3, and evaluates the models on the testing cohort (*n* = 60). Model performance is quantified with identification rate and localization error and compared to other prominent approaches in the literature using the same testing cohort.

## 3 RESULTS

### 3.A A CNN for spinal canal segmentation (preprocessing)

The mean (±standard deviation) 2D distance between the predicted and ground truth spinal canal centroids ranged from 0.343 ± 0.516 mm to 0.426 ± 2.568 mm for the five models made via cross‐validation. Using the best performing model from cross‐validation, the mean (±standard deviation) distance from ground truth centroid to predicted centroid over all CT slices (24,631 slices from 330 patients) in the spinal canal final test set was 0.339 ± 0.299 mm. Only one image slice had distance greater than 5 mm. The centroids of these contours obtained submillimeter accuracy and were appropriate as a preprocessing tool in our vertebral labeling approach. This model performs robustly in contouring the spinal canal in the presence of surgical implants, intravenous contrast, and variable patient orientation. In addition, this model successfully segmented the spinal canal of every patient in the MICCAI 2014 dataset (*n* = 302), demonstrating its robustness.

### 3.B Training and cross validation for vertebral level labeling using simulation CT images

The percentage of patients having 100% vertebral labeling accuracy indicates how well the model performs. Because vertebral bodies on the superior and inferior ends of the CT scan may be partially visible, we considered excluding any labels which were placed on partially visible vertebral bodies. However, by removing three vertebral bodies from the distal ends of the CT scan, we found a large percentage of patients exhibited 100% accuracy (referred to as “% passing” in this work) in the cross‐validation dataset. This was justifiable, as radiotherapy targets for spine palliation are centered about the midpoint of the patient CT. We refer to this portion of the spine as the “clinically viable region” of the CT scan. Thus, based on this criterion, the minimum number of vertebral bodies that can be in any CT scan for this measure (“% passing”, the percentage of patients with all vertebral bodies correctly labeled) to be useful for the evaluation of vertebral labeling performance is seven, equating to about 150 mm or sixty 2.5‐mm CT slices.

**Fig. 3:**
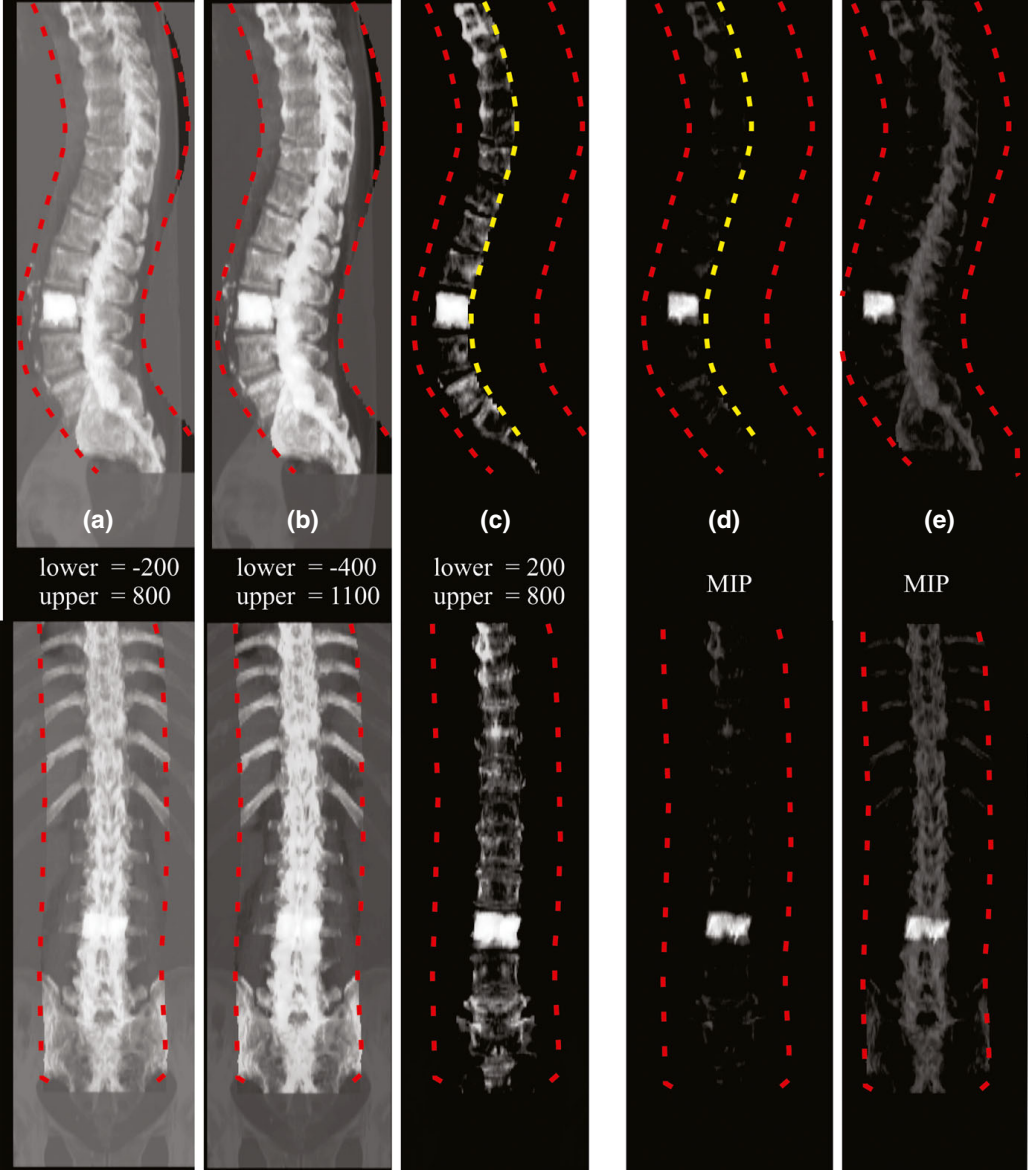
CIP and MIP image pairs. Custom (a–c) and maximum (d–e) intensity projection image pairs depicting sagittal (top row) and coronal (bottom row) views. “Lower” and “upper” indicate bounds for CT number normalization before application of CIP formation formula in 2.C.1. Red dashed lines indicate VOI boundaries formed from the spinal canal. Yellow dashed lines indicate the boundary further constraining the VOI at the midpoint of each axial slice from the spinal canal (thus excluding posterior processes from the VOI). In this patient, from the cross‐validation dataset, a lumbar vertebroplasty is present and creates a high‐intensity region in the IP images. [Color figure can be viewed at wileyonlinelibrary.com]

When using CIP image pairs, we determined that training and evaluating X‐Net using only 3 of the 96 intensity augmentations per patient produced smaller localization error, higher vertebral body identification rate, and higher % passing when compared with using 1, 2, 4, 8, 16, or 96 CIP image pairs as inputs for X‐Net. We selected these three CIP image pairs based on the modal frequency of the most valuable CIP image pair across all vertebral bodies. Using 3 rather than 96 CIP image pairs per patient saves time in the preprocessing and inference steps, reducing the time it takes to automatically label all vertebral levels. One of three of these CIPs had posterior processes removed as described in section 2.C.1. To further clarify, only these three CIP image pairs (and their augmentations) were used during training [Figs. 3(a)‐3(c)]. For inference, raw predictions from each CIP were added together and thresholded (>1.0, determined through optimizing the threshold on the cross‐validation dataset).

Identification rates (all‐regions) were higher on average (1.5%, minimum = 0.8, maximum = 2.6) for a CIP‐based approach vs an MIP‐based approach. Localization errors (all‐regions) were lower on average (0.1 mm, minimum = 0.1 mm, maximum = 0.3 mm) for a CIP‐based approach vs an MIP‐based approach. In addition, %passing was markedly higher on average (8%, minimum = 4%, maximum = 12%) for a CIP‐based approach vs an MIP‐based approach. One possible explanation for poorer MIP performance is the domination of pixel intensity by surgical implants or other high CT number regions. Since MIP image pairs were normalized to the maximum signal intensity, pixels from the vertebroplasty in [Figs. 3(d)‐3(e)] dominate and de‐emphasize lower intensity pixels of surrounding bony anatomy.

Thus, all models were retrained using these specific CIP image pairs and all results below were calculated using a CIP‐based approach with three CIP image pairs per patient. The X‐Net model resulting from the fifth cross validation split was the best performing model from cross validation in terms of % passing with 149 of 162 (92%) of patients labeled correctly within the clinically viable region (Table III). Thus, we used the X‐Net model from Split 5 to optimize the method to detect irregular intervertebral spacing and calculate final test set results.

**Table III:** Mean class‐specific cross‐validation results for X‐Net.

	Split 1			Split 2			Split 3			Split 4			Split 5		
	IR	Mean	Std	IR	Mean	Std	IR	Mean	Std	IR	Mean	Std	IR	Mean	Std
All Regions	89.6	2.4	1.8	92.7	2.3	1.8	**95.2**	2.2	1.7	94.8	2.2	1.6	94.8	**2.0**	**1.6**
Cervical	97.9	2.5	1.5	96.3	2.3	1.5	**98.5**	2.3	1.4	96.2	2.4	1.5	97.6	**2.1**	**1.3**
Thoracic	91.8	2.3	1.7	94.2	2.2	1.7	**96.6**	**1.9**	1.5	95.6	2.1	**1.5**	94.5	2.1	1.5
Lumbar	90.1	2.4	2.1	86.8	2.2	1.8	89.7	2.0	**1.5**	**92.2**	**2.1**	1.8	90.3	2.1	1.8
Sacral	46.2	3.7	2.7	85.2	3.6	2.3	89.7	3.6	2.7	91.9	**3.3**	**1.9**	**97.8**	3.5	2.0
Cases	162			161			159			158			162		
Vertebrae	2,687			2,644			2,555			2,698			2,665		
% Pass	87%			90%			89%			89%			92%		

IR, Mean class identification rate [%]; Mean, Mean class localization error [mm]; Std, standard deviation of class localization error [mm]. The highest scoring metrics across all models are in bold.

### 3.C Study of model performance vs CT scan length

**Fig. 4:**
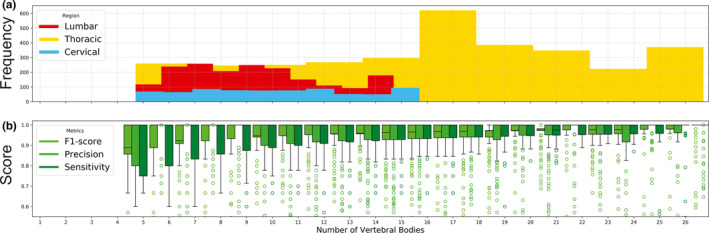
(a) The frequency of cropped CIP image pairs, grouped by the number of vertebrae per cropping. Lumbar (red), thoracic(yellow), and cervical (blue) indicate if the cropped scan was centered upon lumbar, thoracic, or cervical regions. (b) F_1_ score (lime green), precision (green), and sensitivity (dark green) as a function of the number of vertebral bodies within each cropping (irrespective of scan type). [Color figure can be viewed at wileyonlinelibrary.com]

Using the method described in section 2.C.1 (depicted in Figs. 2(b) and 2(c) we created 5,996 pseudo‐CIP image pairs from 162 patient scans. Increasing the CT scan length increased F_1_ score, precision, and sensitivity irrespective of scan length (Fig. 4). When resulting predictions were grouped by scan type (whether the cropped image was centered on a cervical, thoracic, or lumbar vertebral body) (Table 4), metrics increased with increasing scan length and the standard deviation of each metric decreased except for lumbar centered scans. CT scans that completely encompassed the patient's spine 1) captured relevant anatomic information (e.g., additional lumbar [L6] or thoracic [T13] vertebrae) that may otherwise cause incorrect vertebral labeling and 2) had the tightest interquartile ranges for F_1_ score, and sensitivity (Fig. 4.).

**Table IV:** Mean F_1_‐score, precision, and sensitivity grouped by scan type.

Vertebrae per scan	F_1_ score			Precision			Sensitivity		
	C	T	L	C	T	L	C	T	L
<10	93.2 ± 7.0	88.3 ± 12.4	94.6 ± 10.4	96.6 ± 9.2	91.5 ± 16.8	94.7 ± 12.4	90.1 ± 7.8	87.4 ± 8.7	95.6 ± 9.4
10‐20	94.7 ± 6.2	93.3 ± 11.0	94.4 ± 13.4	96.8 ± 9.3	93.3 ± 14.4	92.6 ± 16.3	93.6 ± 4.9	94.6 ± 6.5	97.8 ± 7.8
>20	n/a	96.1 ± 8.1	n/a	n/a	95.5 ± 9.8	n/a	n/a	97.2 ± 7.5	n/a

C, Cervical; T, Thoracic; L, Lumbar.

### 3.D Optimizing the detection of irregularly spaced vertebral bodies

**Fig. 5:**
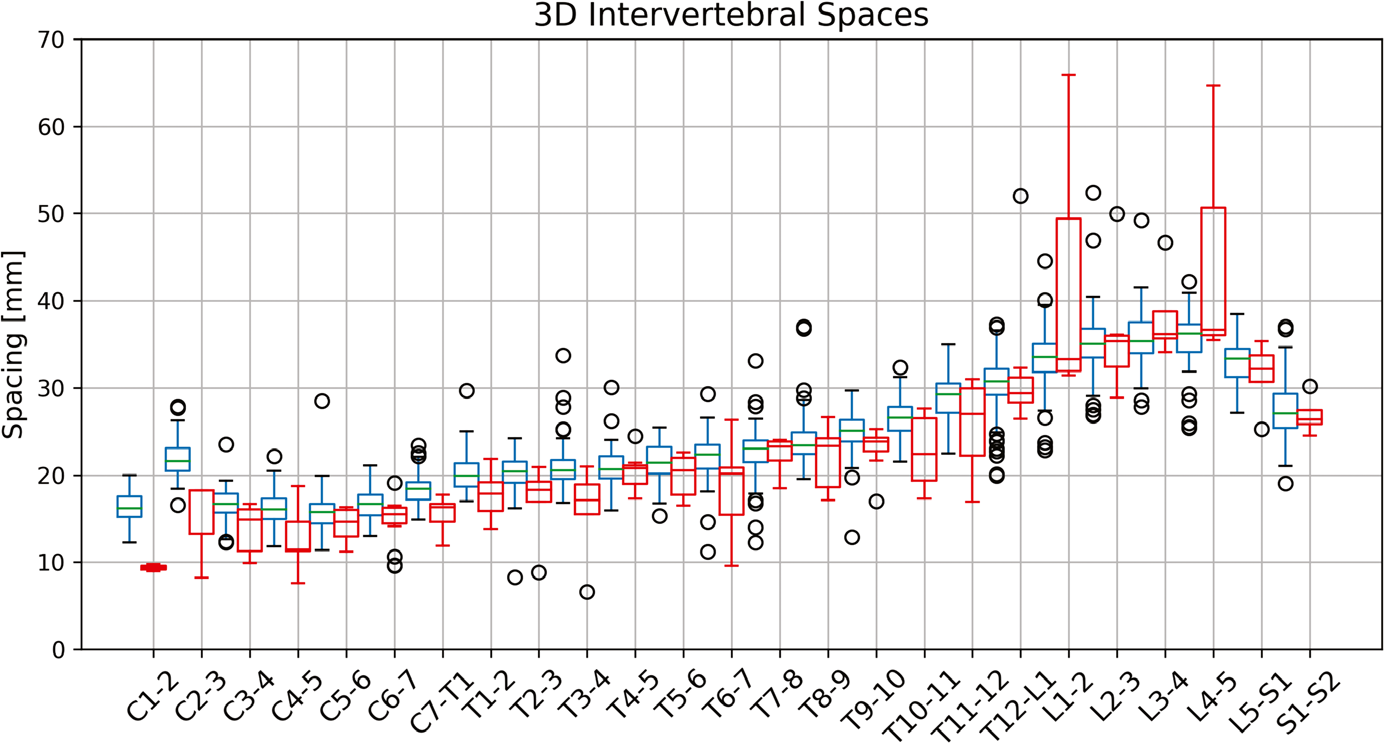
Intervertebral spacing distribution in the best performing cross‐validation split using X‐Net. This spacing is the 3D distance from one vertebral body to the next in millimeters. Box plots in red and blue indicate junctions where vertebral bodies were incorrectly or correctly labeled, respectively. [Color figure can be viewed at wileyonlinelibrary.com]

**Fig. 6:**
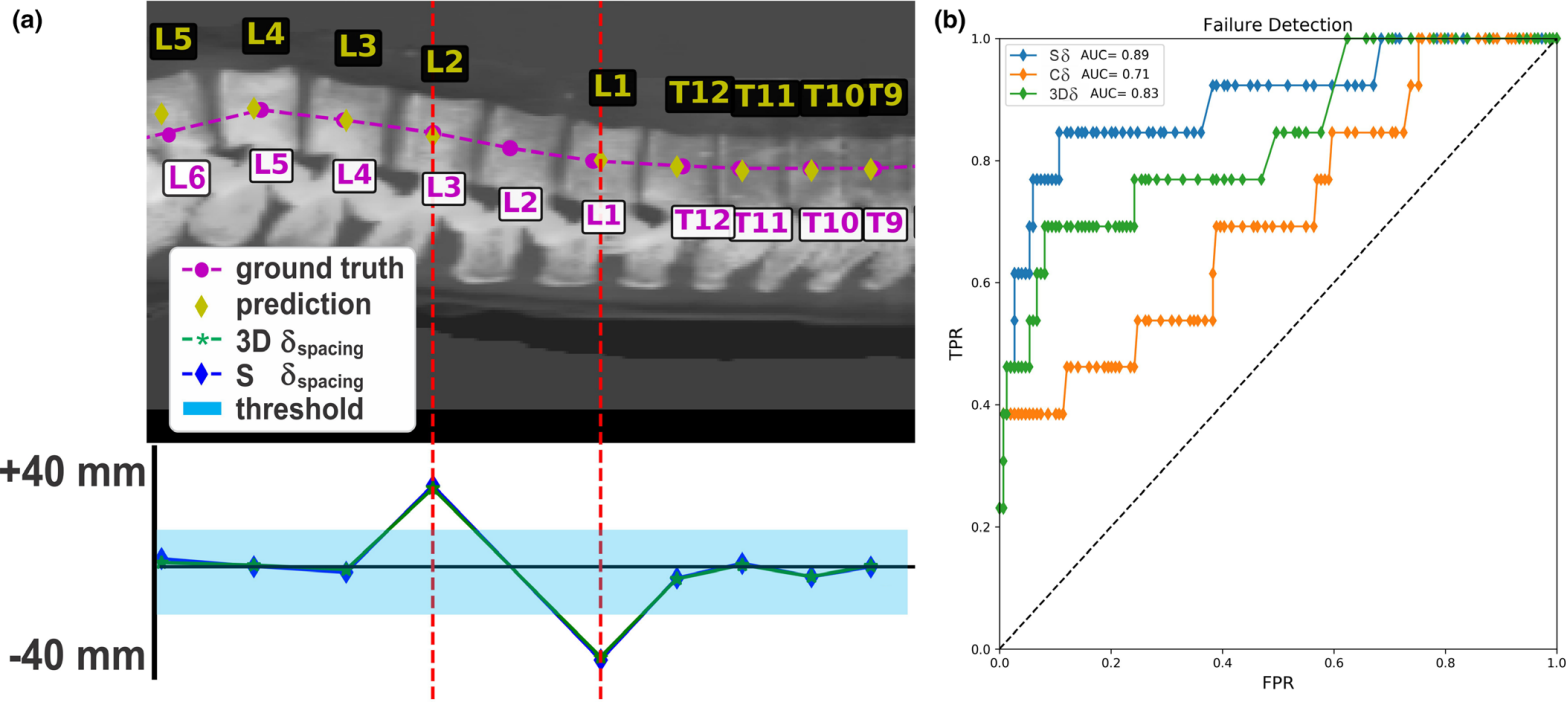
Optimization of the method to detect labeling failures. An example of a sagittal CIP prediction featuring a large rate of change in vertebral body spacing detection (a) and ROC curve (b). Blue, green, and orange plots are sagittal, 3D, and coronal δ plots, respectively. A large δ at L3‐L1 exists. This patient has an additional lumbar vertebrae (L6). The presence of a large gap between predicted lumbar centroids was observed when more than five lumbar exist. [Color figure can be viewed at wileyonlinelibrary.com]

Depending on the vertebral level, large and small intervertebral spaces predicted by X‐Net can lie far outside the median for correctly and incorrectly labeled vertebral bodies (Fig. 5). By examining the rate of change in spacing from one vertebral body to the next (δ_space_), sudden changes in intervertebral spacing can be detected and examined using only predictions of X‐Net on the CIP image pairs. There were 13 of 162 patients in the best performing cross validation split whose predictions of vertebral level caused a labeling failure within the clinically viable region. An example of a labeling failure in the lumbar region is depicted in [Fig 6(a)]. By varying the δ threshold region [shaded blue region in Fig. 6(a)], we performed an ROC analysis of the method to detect irregularly spaced vertebral bodies. Using the sagittal δ data [blue diamonds in Fig. 6(b); AUC = 0.89] and setting the threshold for detection to be 10.1 mm, we achieved sensitivity and specificity of 85% and 94%, respectively, for detecting labeling failures.

### 3.E Final testing of vertebral level labeling and irregular spacing detection on radiotherapy simulation CT scans

#### 3.E.1 Model Performance Comparisons of X‐Net Ensemble, X‐Net, and Btrfly Net

The X‐Net Ensemble had the highest performing metrics (identification rate; localization error) for all regions (94.2%; 2.2 mm), cervical (97.0%; 2.3 mm), thoracic (94.9%; 2.1 mm), and lumbar (88.4%; 3.6 mm) vertebrae within the final test set (Table V). X‐Net alone had the lowest localization error for sacral vertebrae in the final test set (3.5 mm) (Table V). Within the normative group of the final test set (n = 74), the X‐Net Ensemble had the highest performing metrics (identification rate; localization error) for all regions (96.9%; 2.0 mm), thoracic (96.6%; 1.9 mm), lumbar (93.4%, 1.9 mm), and sacral (100.0%; 3.3 mm) vertebrae (Table VI). X‐Net alone had the highest localization error for cervical vertebrae within the normative group of the final test set (99.6%) (Table VI). Within the outlier group of the final test set (*n* = 20), the X‐Net Ensemble had the highest identification rates for all regions (82.6%), cervical (86.3%), thoracic (86.6%), and lumbar (70.9%) vertebrae (Table VII). Btrfly Net had the highest localization error for sacral vertebrae within the outlier group of the final test set (80.2%) (Table VI).

**Table V:** Results for X‐Net, an X‐Net ensemble, and Btrfly Net in the final test set (*n* = 94).

Metric	X‐Net Ensemble			X‐Net			Btrfly Net		
	IR	Mean	Std	IR	Mean	Std	IR	Mean	Std
All Regions	**94.2**	2.2	1.8	92.4	2.3	1.8	90.5	3.4	2.5
Cervical	**97.0**	2.3	1.7	96.0	2.4	1.5	90.8	3.5	2.1
Thoracic	**94.9**	2.1	1.4	93.6	2.2	1.6	91.5	3.5	2.5
Lumbar	**88.4**	2.2	2.9	83.4	2.3	2.3	86.4	2.8	2.5
Sacral	**94.4**	3.6	2.9	**94.4**	3.5	2.6	94.2	4.2	2.4

IR, Mean class identification rate [%]; Mean, Mean class localization error [mm]; Std, standard deviation of class localization error [mm]. The highest accuracy per region is shown in bold.

**Table VI:** Results for X‐Net, an X‐Net ensemble, and Btrfly Net in the normative group (*n* = 74) of the final test set.

Metric	X‐Net Ensemble			X‐Net			Btrfly Net		
	IR	Mean	Std	IR	Mean	Std	IR	Mean	Std
All Regions	**96.9**	2	1.3	96.7	2.2	1.4	94.8	3.3	2.3
Cervical	99.2	2.2	1.3	**99.6**	2.4	1.5	94.4	3.6	2.1
Thoracic	**96.6**	1.9	1	96.3	2.1	1.3	94.4	3.5	2.4
Lumbar	**93.4**	1.9	1.2	91.6	1.9	1.0	95.2	2.6	2.1
Sacral	**100.0**	3.3	2.4	**100.0**	3.4	2.6	98.1	4.0	2.2

IR, Mean class identification rate [%]; Mean, Mean class localization error [mm]; Std, standard deviation of class localization error [mm]. The highest accuracy per region is shown in bold.

**Table VII:** Results for X‐Net, an X‐Net ensemble, and Btrfly Net in the outlier group (*n* = 20) of the final test set.

Metric	X‐Net Ensemble			X‐Net			Btrfly Net		
	IR	Mean	Std	IR	Mean	Std	IR	Mean	Std
All Regions	**82.6**	3.2	3.2	74.3	3.2	3.0	72.0	3.9	3.2
Cervical	**86.3**	2.9	2.9	77.8	2.6	1.6	72.4	3.0	1.8
Thoracic	**86.6**	2.9	2.4	80.4	3	2.4	77.8	4.0	3.2
Lumbar	**70.9**	3.5	4.4	54.8	4.1	4.9	54.4	3.9	3.8
Sacral	74.6	5.1	4.1	74.6	4.0	2.2	**80.2**	4.9	3.2

IR, Mean class identification rate [%]; Mean, Mean class localization error [mm]; Std, standard deviation of class localization error [mm]. The highest accuracy per region is shown in bold.

**Fig. 7:**
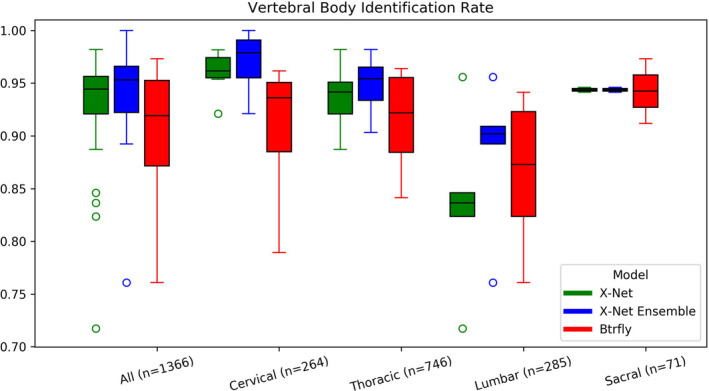
Vertebral body identification rates for X‐Net (green), the X‐Net ensemble (blue), and Btrfly Net (red) for all vertebral bodies within the final test set (*n* = 94). [Color figure can be viewed at wileyonlinelibrary.com]

**Fig. 8:**
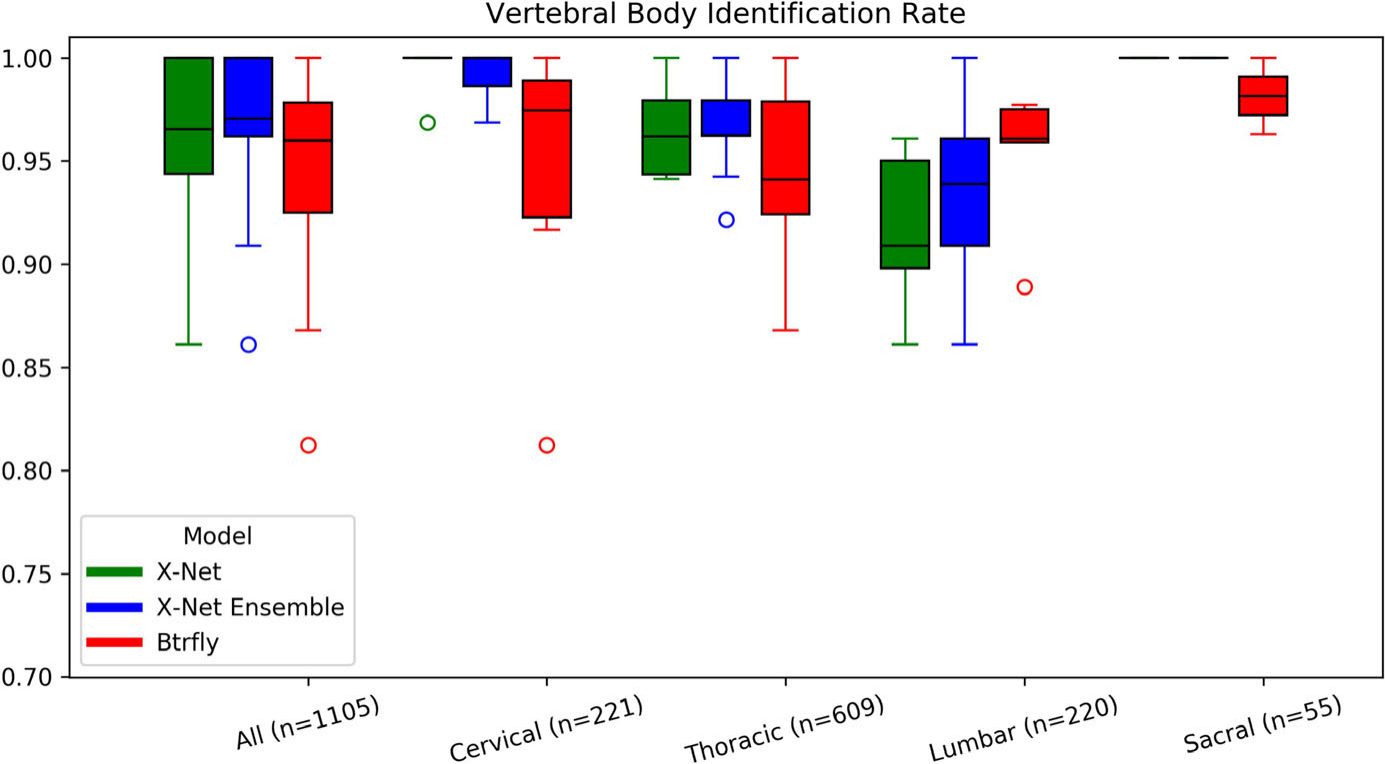
Vertebral body identification rates for X‐Net (green), the X‐Net ensemble (blue), and Btrfly Net (red) for vertebral bodies in the normative group within the final test set (*n* = 74). [Color figure can be viewed at wileyonlinelibrary.com]

**Fig. 9:**
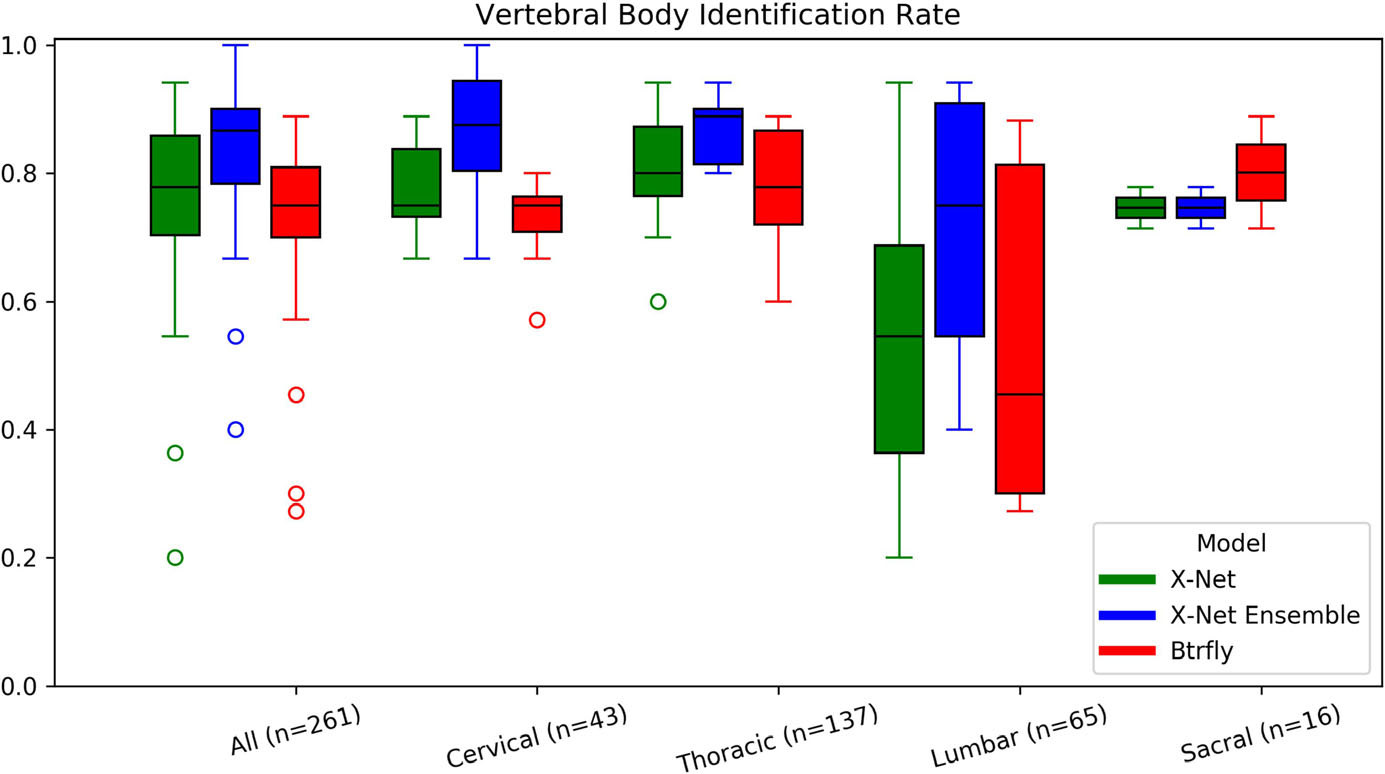
Vertebral body identification rates for X‐Net (green), the X‐Net ensemble (blue), and Btrfly Net (red) for vertebral bodies in the outlier group within the final test set (*n* = 20). [Color figure can be viewed at wileyonlinelibrary.com]

**Fig. 10:**
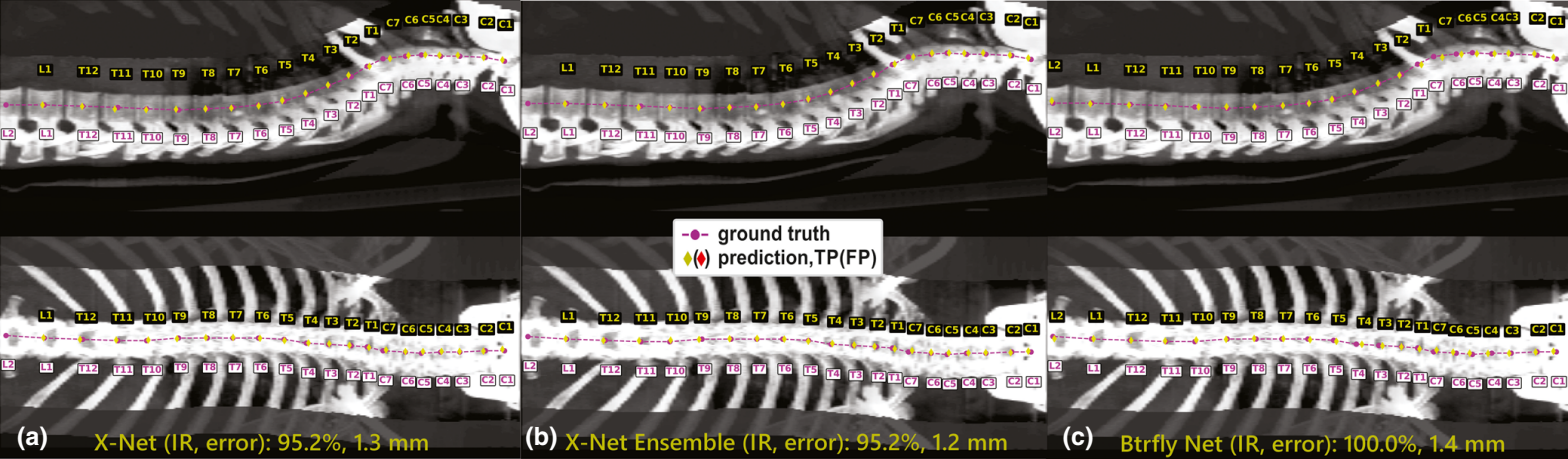
Model predictions of vertebral level on a normative patient with 21 vertebral bodies spanning C1‐L2. (a) X‐Net, (b) X‐Net ensemble, and (c) Btrfly Net predictions show TPs in yellow diamonds and FPs in red diamonds. Ground truth predictions are shown in magenta circles. The clinically viable region spanned T11‐C4, and all three predictions were 100% accurate in this region. IR, identification rate; error, localization error. [Color figure can be viewed at wileyonlinelibrary.com]

Median identification rates for the X‐Net Ensemble are the highest for a majority of vertebral regions for the final test set (Fig. 7) as well as normative (Fig. 8) and outlier (Fig. 9) groups within the final test set. Mean and median identification rates and localization errors from Btrfly Net were inferior to identification rates and localization errors from X‐Net alone and the X‐Net Ensemble (TablesV, VI, VII, Figs. 7, 8, 9) except for identification rate of sacral regions within the normative group of the final test set Fig. 10.

**Fig. 11:**
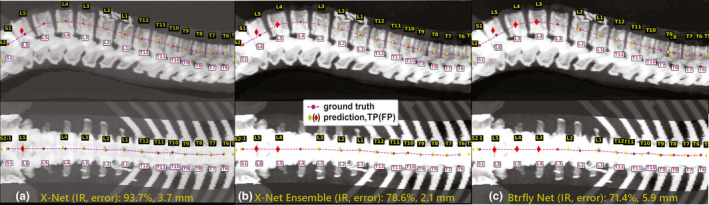
Model predictions of vertebral levels for a patient with an extra lumbar body (L6) in the outlier group within the final test set. (a) X‐Net, (b) X‐Net ensemble, and (c) Btrfly Net predictions TPs in yellow diamonds and FPs in red diamonds. Ground truth predictions are shown in magenta circles. High pixel numbers above L1‐L2 were due to the presence of nephrectomy surgical clips. In sagittal views of (a),(b), and (c) large spaces were left at L5, L4, and L3, respectively. IR, identification rate; error, localization error. [Color figure can be viewed at wileyonlinelibrary.com]

When vertebral level predictions from X‐Net, X‐Net Ensemble, or Btrfly Net predicted vertebral level on a CT scan with more than five lumbar vertebral bodies, a gap spanning a lumbar vertebral space was left on both sagittal and coronal CIPs [Figs. 11(a)‐11(c)]. All three models label the vertebral body immediately superior to S1 as L5 for all patients with irregular lumbar vertebral anatomy.

#### 3.E.2 Detection of labeling failures from X‐Net, X‐Net Ensemble, and Btrfly Net

After vertebral labels were predicted using X‐Net, X‐Net Ensemble, and Btrfly Net, the number of patients from the final test set (*n* = 94) that exhibited labeling failures was 10, 6, and 34, respectively. The sensitivity and specificity of the method to detect these labeling failures was 80% and 89%, 67% and 95%, and 90% and 53%, when levels were predicted with X‐Net, X‐Net Ensemble, and Btrfly Net, respectively.

The X‐Net Ensemble paired with the method to detect irregular spacing performed with the highest sensitivity to catching these errors and also exhibited the lowest number of FN's (2)—one in the normative group and one in the outlier group. The FN in outlier group was a patient in the prone position. The FN in the normative group belonged to a CT scan which was situated between anatomical landmarks in the thoracic spine (T12‐T3), all vertebral labels were systematically off by one. Two of four FP's were attributed to large vertebral spaces from patients with surgical hardware that introduced atypical intervertebral spacing. Two other FP's were from CT scans of patients with atypical lumbar counts (when labeling failures were outside of the clinically viable region).

**Fig. 12:**
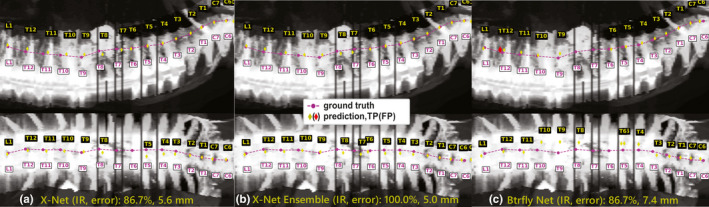
Model predictions of vertebral level on patient with extensive surgical implants (spanning L1‐T4) in the outlier group within the final test set. (a) X‐Net, (b) X‐Net ensemble, and (c) Btrfly Net predictions show TPs in yellow diamonds and FPs in red diamonds. Ground truth predictions are shown in magenta circles. IR, identification rate; error, localization error. [Color figure can be viewed at wileyonlinelibrary.com]

All six patients with surgical implants (six of twenty outlier group patients within the final test set) exhibited 100% accuracy within the clinically viable region when the X‐Net Ensemble predicted vertebral level (Table VIII, Fig. 12).

**Table VIII:** % Pass scores for X‐Net, the X‐Net Ensemble, and Btrfly Net.

	X‐Net Ensemble		X‐Net		Btrfly Net	
	# fail	% Pass	# fail	% Pass	# fail	% Pass
All (*n* = 94)	6	94	10	89	18	81
Normative (*n* = 74)	2	97	1	99	7	91
Outlier (*n* = 20)	4	80	9	55	11	45

# fail, number of patients with vertebral level predictions that fail within the clinically viable region; %Pass, percentage of patients with 100% accuracy within the clinically viable region (i.e., excluding the three most superior and inferior vertebral bodies).

Due to the fact that vertebral level predictions left a large gap between vertebrae when the CT scan contained the presence of an additional vertebra (i.e., T13 or L6), the method to detect irregular intervertebral spaces was able to flag four of four patients which possessed a sixth lumbar vertebral body and the one patient who possessed a thirteenth vertebral body. The method to detect irregular spacing did not detect when the patient only had four lumbar vertebral bodies. Thus, the method to detect irregular spacing, when tested on predictions from the X‐Net Ensemble, could detect five of six patients with irregular vertebral anatomy.

### 3.F Comparison to previous work

When the X‐Net Ensemble was trained and tested on the MICCAI 2014 dataset, it achieved an identification rate and localization error (all regions) of 86.8% and 3.8 mm (Table IX, **“Proposed”**). Our simple approach applying transfer learning a) loaded all five X‐Net models used in cross validation on our radiotherapy simulation CT cohort (*n* = 803), b) cut learning rate by a factor of 100–2e^−6^, c) trained for two epochs (with no layers frozen), d) ran predictions from each model on the test set (*n* = 60), and e) ensembled the prediction results (median of 3D centroid predictions). For comparison, this same transfer learning approach (also including identical loss, data splits, choice of CIP images, and augmentation to use for the initial training of X‐Net) was performed for Btrfly Net and is also featured in the table below (Table IX, “Btrfly Net_TL_”). Using X‐Net, identification rates and localization errors from this transfer learning approach achieved state‐of‐the‐art results over previous approaches which also include training data from outside cohorts (Table IX, **“Proposed_TL_”**).

**Table IX:** Performance of X‐Net compared with previous approaches on the same testing dataset.

Author	Identification rate [%]					Localization Error [mm], mean (standard deviation)				
	All	C	T	L	S	All	C	T	L	S
H. Chen et al.^27^	84.2	91.8	76.4	88.1	n/a	8.8(13.0)	5.1 (8.2)	11 (17)	8.2 (8.6)	n/a
Yang et al.^28^	85.0	92.0	81.0	83.0	n/a	8.6 (7.8)	5.6 (4.0)	9.2 (7.9)	11 (11)	n/a
Liao et al.^18^	88.3	**95.1**	84.0	92.2	n/a	6.5 (8.6)	4.5 (4.6)	7.8 (10.2)	5.6 (7.7)	n/a
Sekuboyina et al.^16^	86.7	89.4	83.1	92.6	n/a	6.3 (4.0)	6.1 (5.4)	6.9 (5.5)	5.7 (6.6)	n/a
Sekuboyina et al.^16^	87.7	89.2	85.8	92.9	n/a	6.4 (4.2)	5.8 (5.4)	7.2 (5.7)	5.6 (6.2)	n/a
Sekuboyina et al.^16^	88.5	89.9	86.2	91.4	n/a	6.2 (4.1)	5.9 (5.5)	6.8 (5.9)	5.8 (6.6)	n/a
McCouat et al.^21^	85.5	90.6	79.8	92.0	n/a	5.6 (7.1)	3.9 (5.3)	6.6 (7.4)	5.4 (8.7)	n/a
J. Chen et al.^29^	88.0	n/a	n/a	n/a	n/a	7.1 (7.1)	n/a	n/a	n/a	n/a
Jakubicek et al.^22^	90.9	n/a	n/a	n/a	n/a	5.1 (4.0)	4.21 (**0.6**)	5.3 (**1.3**)	6.6 (**0.6)**	n/a
Qin et al.^20^	89.0	90.8	86.7	89.7	**96.9**	2.9 (5.8)	**2.2** (5.6)	3.4 (6.5)	2.9 (4.3)	**2.2** (**2.7**)
Y. Chen et al^30^	**94.7**	89.5	**95.3**	**100**	n/a	**2.6** (3.2)	2.5 (3.7)	**2.6** (3.3**)**	**2.2** (1.8**)**	n/a
**Proposed**	86.8	94.0	80.1	91.1	90.6	3.8 (**2.9**)	3.3 (2.3)	3.9 (3.0)	3.7 (3.2)	5.8 (3.7)
Yang et al.^28^ *	90.0	93.0	88.0	90.0	n/a	6.4 (5.9)	5.2 (4.4)	6.7 (6.2)	7.1 (7.3)	n/a
Btrfly Net_TL_ *	87.1	86.6	86.5	90.4	**84.4**	4.1 (2.8)	3.7 (2.4)	4.5 (3.0)	3.8 (2.7)	**4.9** (**2.8)**
Proposed_TL_ *	**91.3**	**93.6**	**90.6**	**92.5**	**84.4**	**3.3 (2.7)**	**3.0 (2.0)**	**3.5 (2.8)**	**2.8 (2.4)**	5.9(3.8)

All, All regions; C, Cervical, T, Thoracic; L, Lumbar; S, Sacral. *Represents approaches which use data in addition to that provided by the MICCAI 2014 dataset. ProposedTL = X‐Net with transfer learning on a cohort of 803 patients; Yang et al.^28^ *indicates the addition of patients outside the MICCAI 2014 dataset. All approaches listed in the table are 3D‐based except Btrfly Net^16^ and X‐Net which are projection‐based. Scores from Btrfly Net without the addition of a general adversarial network are featured in the topmost row of approaches by Sekuboyina *et al*. The highest scoring metrics across all models (*with and without additional data) are in bold.

## 4 DISCUSSION

X‐Net is a unique architecture that we designed based on VNet^13^ and other methods^12^ used to automatically label vertebral level in CT images. Our patient cohort contained CT scans with significant bony metastasis burden, irregular vertebral body counts, radiotherapy‐specific patient positioning, and acquisition parameters (e.g., field of view, slice thickness) specific to simulation CTs for radiotherapy. In addition to training a robust spinal canal segmentation architecture, our custom intensity projection images (constrained by a spinal canal VOI) combine both mean and maximum values from the patient CT to increase performance compared to approaches using maximum intensity projection images alone. Previously, Sekuboyina et al. studied the impact of using a VOI constrained AIP vs VOI constrained MIP for spinal localization in Btrfly Net and found the MIP was preferable.^16^ However, these authors found that the standard deviation of the localization when using AIP was similar to that of the MIP. Although our CIPs were designed from a purely qualitative perspective, CIP‐based approaches in this work offer a quantifiable benefit over MIP‐based approaches and may better emphasize bony anatomy when high‐intensity pixels are present from surgical implants (Fig. 3). When tested on palliative radiotherapy CT scans of patients with metastatic disease, surgical implants, or other defects or anatomic irregularities, X‐Net automatically labels vertebral levels (C1‐S2) with a high identification rate (94%) and small localization errors (2.2 ± 1.8 mm). This approach identified vertebral levels correctly within the clinically viable region for 97% of patients when CT scans were pre‐screened for outliers, and for 94% of patients when CT scans were not pre‐screened for outliers. In addition, our automated vertebral labeling approach performed accurately when surgical implants were present, and the method to detect irregular intervertebral spacing flagged five of six patients with abnormal vertebral anatomy. Because this approach can preprocess CT images and predict vertebral levels in under 3 min, it could be used in the treatment planning process to expedite the patient care path for those needing rapid palliation.

To expedite the patient care path, some authors have used cone beam CT or diagnostic CT images rather than simulation CT images to create the treatment plan.^27^, ^28^ Others have used simulation CT images for treatment planning but implemented different strategies to increase care path efficiency.^6^, ^29^, ^30^, ^31^ Despite the choice of imaging or optimized care path, localization of the correct target by labeling vertebral levels is required. In all of these instances, vertebral levels were labeled manually. Some of the first methods of automating vertebral labeling were a 2D approach used by Peng et al.^32^ and a 3D approach used by Schmidt et al.^33^ with MR images. They used either disc detection methods involving convolutions or probabilistic graph models paired with a multiclass classifier. Later, Klinder et al.^34^ provided a model‐based approach to detecting, identifying, and segmenting vertebrae in CT image volumes_._ Glocker and colleagues followed this by using regression forests and Markov models to identify vertebral levels, and they improved upon such work by using classification forests to increase vertebral level identification rate to over 70% and decrease median localization error to 8.8 mm^14^, ^15^_._ The dataset used by Glocker et al. contains CT diagnostic image volumes with tightly cropped fields of view, postoperative implants, and random pathologies. Since this dataset became publicly available during the MICCAI 2014 CSI Workshop, it has served as a test benchmark for subsequent studies of automatic vertebral labeling using CT scans. Although investigators have made progress in improving vertebral identification rate with machine learning‐based models using hand‐crafted features, significant gains in identification rate and reductions in localization error soon resulted from the advent of deep learning.^17^, ^18^, ^35^, ^36^, ^37^ One example by Sekuboyina et al.^12^, ^16^ devised a 2D deep learning approach using a dual‐input architecture termed “Btrfly Net”, which used preprocessed MIP images from coronal and sagittal views of patient CT scans as inputs. This new approach was benchmarked against past efforts as well as U‐Net,^38^ a well‐known deep learning architecture. Recent work by Chen et al^39^ uses a combination of 3D, 2D, and attention‐based globally refining modules to automatically localize and identify vertebrae, bringing identification rate to 88% and mean localization error to 7.1 mm. Jakubicek *el al*. combined a set of deep learning algorithms using spinal detection, cord tracking, intervertebral disc localization, and CNN‐based classification to bring identification rate to 90.9% and mean localization error to 5.1 mm^22^. This unique combination of architectures was also tested upon a dataset of diagnostic CT images containing bone metastases (*n = *49) and performed with high identification rate (95%) and small localization error (2.34 ± 0.84 mm), but requires the presence of intervertebral discs which can be absent when metastatic disease degrades portions of the spine. Qin et al. introduced a residual block‐based CNN with separate branches for localization and classification of vertebral bodies and reported increases in identification rate for thoracic and sacral regions of the spine upon the MICCAI 2014 dataset.^20^ Finally, Chen et al. also made significant improvements in performance on this dataset by using an FCN and Hidden Markov Model, bring identification rate and localization error to 95% and 2.6 mm, respectively.^40^

Identification rate and localization error from X‐Net's predictions on this dataset show that our approach ranks among some of the most accurate approaches thus far (Table IX). Furthermore, our transfer learning approach using a pre‐trained X‐Net Ensemble (with knowledge from a patient cohort of 803 CT scans) demonstrated state‐of‐the‐art performance for all regions of the spine, which outranks past approaches that supplement the training data with 1,000 additional CT scans by Yang et al.^17^ When the same transfer learning approach was applied to Btrfly Net, identification rates still did not surpass results from X‐Net except for those in sacral regions (Table IX). Although effective, our transfer learning approach may be further improved by freezing lower level layers of the network and fine‐tuning higher‐level features.

Our work contributes to prior research described above in that it introduces a deep learning architecture that can automatically label vertebral level in CT scans with high identification rate and low localization error on multiple patient cohorts. X‐Net's dual‐input, dual‐output framework was inspired by work from Sekuboyina et al.^16^ In terms of X‐Net's inner structure, various segmentation architectures (e.g., UNET^38^) have incorporated maxpooling and upsampling operations between stages, whereas X‐Net replaces such operations by down and up convolutions, respectively. This was motivated by work from Milletari et al.,^13^ and subsequently Schreier et al.,^41^ where kernels with stride 2 are used in convolutional layers in replacement of maxpooling layers or upsampling layers. Others have shown that replacing max pooling for downsampling convolutional layers provides an improvement in terms of training stability, which was another rationale for this change.^42^ In addition, X‐Net was designed to have residual blocks at each convolutional stage. The use of residual connections was motivated by works from He et al.^43^ and Szegedy et al.^44^ and is used in each stage to merge features across multiple convolutional layers and increase gradient flow. Such residual connections have been shown to be beneficial to medical image segmentation when implemented in encoder‐decode networks.^41^ In order to increase network depth and information gain, we designed X‐Net to have four stages before features are concatenated into shared arms of the architecture. As seen by Zhou et al., increasing depth while incorporating specialized connections between convolutional layers allows for increased performance for UNET type architectures.^45^ Lastly, we used PReLU^46^ activations instead of ReLU activations. This was done as means to prevent ReLU‐related neuronal death.^47^ Thus, compared with Btrfly Net, X‐Net has more convolutional stages, more convolutions per stage, residual connections at each stage, convolutional operations in place of pooling operations, and PReLU activations in place of ReLU. These structural choices resulted in an architecture capable of delivering improved results on multiple datasets via an end‐to‐end approach. Although our approach is end‐to‐end trainable, a limitation to this work is our choice to not enhance X‐Net with adversarial training, a known technique for increasing accuracy and decreasing localization errors.^16^

Although clinicians may have access to prior multimodality imaging (MRI and/or CT) to verify vertebral levels during treatment planning, X‐Net predictions of vertebral levels obtained upon a CT scan with incomplete anatomical information will yield incorrect results. For example, if the scan of the spine in Fig. 11 were to include only the five most inferior lumbar vertebral bodies, the presence of a sixth lumbar body may go undetected. Additionally, methods of manually counting vertebral levels on CT images require inclusion of the clavicle, xiphoid process, or sternal angle to serve as an anatomical landmark for counting the ribs^5^. In terms of model performance as a function of scan length, increasing the scan length increases the accuracy [Fig. 4(b), Table IV], and is most accurate when the CT scan includes the entire spine.

To further bolster the usefulness of X‐Net and its safe application to radiotherapy treatment planning, we devised our method of detecting vertebral labeling failures based on the rate of change of intervertebral spacing. For patients with vertebral spaces not within the typical median distance (Fig. 5) for each vertebral body junction (e.g., those with vertebral collapse due to pathologic compression and/or expanded junctions from surgical implants), using a fixed distance threshold to flag outlier spaces was impractical. Thus, the rate of change of spacing was more useful than using a fixed distance threshold and can be used to identify vertebral bodies that are too close together or too far apart based on neighboring vertebral spacing. Using this method, we detected all patients with additional lumbar (L6) and additional thoracic (T13) vertebrae in CT scans in the final test set. The typical human spine contains 7 cervical, 12 thoracic, and 5 lumbar vertebral bodies, but these counts vary.^48^, ^49^, ^50^ Because the incidence of abnormal lumbar vertebral anatomy is reported to be about 5.5‐6.6%,^49^, ^50^ this method of identifying irregularly spaced vertebral bodies serves as a useful check to detect abnormal lumbar counts in all palliative radiotherapy cases. However, a limitation of this work is that our model did not address the distinct classification of transitional lumbosacral vertebrae during training. Thus, the L5‐S1 junction should be verified by a clinician, as 35.6% of the general population may have transitional lumbosacral vertebrae.^49^ In addition, although the method to detect irregular intervertebral spacing arising from atypical anatomy could identify most anatomic variants in the final test set, X‐Net's ability to only label spines with typical anatomy represents a limitation and could be expanded upon in future work.

Although our approach (an X‐Net Ensemble paired with the method to detect irregular intervertebral spacing) performed well, two of the six labeling failures from the normative group exhibited vertebral labeling failures that went undetected. This represents a limitation, as the FN rate must be minimized to make X‐Net a viable and safe treatment planning tool. One solution to decrease the FN rate is to implement pairwise algorithms with independent failure modes. Rhee et al.^25^ and Kisling et al.^51^ have implemented this approach to create anatomic contours for treatment planning purposes.

Avoiding automation bias and improving clinical safety when applying deep learning models to medicine is paramount.^52^ This work, when paired with a secondary vertebral labeling approach and automatic quality assurance can greatly improve efficiency in radiotherapy treatment planning. Once this has been accomplished, we plan to deploy X‐Net clinically using the Radiation Planning Assistant,^25^, ^53^, ^54^, ^55^ a web‐based, fully automated treatment planning platform, in order to provide automatic vertebral labeling services to our clinic and other clinics in need of automatic vertebral labeling.

## 5 CONCLUSIONS

We trained X‐Net, our convolutional neural network, to automatically label vertebral levels from S2 to C1 on palliative radiotherapy CT images and found that an ensemble of X‐Net models had high vertebral body identification rate (94%) and small localization errors (2.2 ± 1.8 mm). In addition, our transfer learning approach achieved state‐of‐the‐art results on a well‐known benchmark dataset with high identification rate (91%) and low localization error (3.3 mm ± 2.7 mm). When we pre‐screened radiotherapy CT images for the presence of hardware, surgical implants, or other anatomic abnormalities prior to the use of X‐Net, it labeled the spine correctly in more than 97% of patients and 94% of patients when scans were not pre‐screened. Automatically generated labels are robust to widespread vertebral metastases and surgical implants and our method to detect labeling failures based on neighborhood intervertebral spacing can reliably identify patients with an additional lumbar or thoracic vertebral body.

## CONFLICTS OF INTEREST

Funding was received in part from Varian Medical Systems and the National Cancer Institute. Multiple authors of this publication are members of the Radiation Planning Assistant team at the University of Texas MD Anderson Medical Center.
